# Machine learning models reveal the critical role of nighttime systolic blood pressure in predicting functional outcome for acute ischemic stroke after endovascular thrombectomy

**DOI:** 10.3389/fneur.2024.1405668

**Published:** 2024-05-09

**Authors:** Dingkang Xu, Peng Qi, Peng Liu, Hongchun Yang, Gengfan Ye, Dezhi Shan, Shixiong Lei, Guozheng Yang, Junqing Ding, Hui Liang, Hui Qi, Daming Wang, Jun Lu

**Affiliations:** ^1^Department of Neurosurgery, Beijing Hospital, National Center of Gerontology, Institute of Geriatric Medicine, Chinese Academy of Medical Sciences, Beijing, China; ^2^Graduate School of Peking Union Medical College, Beijing, China; ^3^Department of Neurosurgery, Peking University Shenzhen Hospital, Shenzhen, Guangdong, China; ^4^Department of Neurosurgery, Ningbo Medical Center Lihuili Hospital, Ningbo University, Ningbo, Zhejiang, China; ^5^Neurology Department, Xiyuan Hospital, China Academy of Chinese Medical Sciences, Beijing, China; ^6^Department of Neurology, Hainan General Hospital, Hainan Affiliated Hospital of Hainan Medical University, Hainan Province Clinical, Medical Center and Hainan Academician Innovation Platform, Haikou, China

**Keywords:** acute ischemic stroke, endovascular thrombectomy, blood pressure, circadian pattern, machine learning

## Abstract

**Background:**

Blood pressure (BP) is a key factor for the clinical outcomes of acute ischemic stroke (AIS) receiving endovascular thrombectomy (EVT). However, the effect of the circadian pattern of BP on functional outcome is unclear.

**Methods:**

This multicenter, retrospective, observational study was conducted from 2016 to 2023 at three hospitals in China (ChiCTR2300077202). A total of 407 patients who underwent endovascular thrombectomy (EVT) and continuous 24-h BP monitoring were included. Two hundred forty-one cases from Beijing Hospital were allocated to the development group, while 166 cases from Peking University Shenzhen Hospital and Hainan General Hospital were used for external validation. Postoperative systolic BP (SBP) included daytime SBP, nighttime SBP, and 24-h average SBP. Least absolute shrinkage and selection operator (LASSO), support vector machine-recursive feature elimination (SVM-RFE), Boruta were used to screen for potential features associated with functional dependence defined as 3-month modified Rankin scale (mRS) score ≥ 3. Nine algorithms were applied for model construction and evaluated using area under the receiver operating characteristic curve (AUC), sensitivity, specificity, and accuracy.

**Results:**

Three hundred twenty-eight of 407 (80.6%) patients achieved successful recanalization and 182 patients (44.7%) were functional independent. NIHSS at onset, modified cerebral infarction thrombolysis grade, atrial fibrillation, coronary atherosclerotic heart disease, hypertension were identified as prognostic factors by the intersection of three algorithms to construct the baseline model. Compared to daytime SBP and 24-h SBP models, the AUC of baseline + nighttime SBP showed the highest AUC in all algorithms. The XGboost model performed the best among all the algorithms. ROC results showed an AUC of 0.841 in the development set and an AUC of 0.752 in the validation set for the baseline plus nighttime SBP model, with a brier score of 0.198.

**Conclusion:**

This study firstly explored the association between circadian BP patterns with functional outcome for AIS. Nighttime SBP may provide more clinical information regarding the prognosis of patients with AIS after EVT.

## Introduction

Acute ischemic stroke (AIS) remains a leading cause of morbidity and mortality worldwide (1), endovascular thrombectomy (EVT) has become one of the standard therapeutic treatments for AIS patients with large vessel occlusion stroke (LVOS) (2, 3). The relationship between blood pressure (BP) and prognosis in patients with AIS is complex with the available evidence suggests that both insufficient and excessive BP are detrimental to the prognosis of patients with AIS (4, 5).

Post-stroke BP fluctuations represent a multifactorial and intricate physiological process (6). Numerous studies have revealed the correlation between preoperative, intraoperative, and postoperative BP levels and clinical outcomes as an crucial clinical factor (7, 8). While prior studies have emphasized the importance of BP in the prognosis of AIS (9), the optimal BP target for AIS patients remains uncertain. Recent investigations suggested that different BP parameters may provide different clinical information for all-cause death and cardiovascular outcomes when considering the BP circadian patterns. Staplin et al. (10) and Yang et al. (11) have found in large-scale population-based cohorts that higher nighttime BP was significantly associated with greater risk of mortality and composite cardiovascular outcomes even after adjustment for other office-based or ambulatory BP measurements.

In recent years, machine learning (ML) has increasingly become a powerful tool in medical research (12, 13), and ML has also shown excellent performance in predicting the prognosis of AIS (14). The aim of this study was to investigate the association between different circadian SBP parameters and prognosis after EVT in AIS patients. The study may provide a unique perspective for a precise understanding of the association between circadian SBP patterns and the prognosis of AIS patients.

## Methods

### Study design and participants

**Figure 1:**
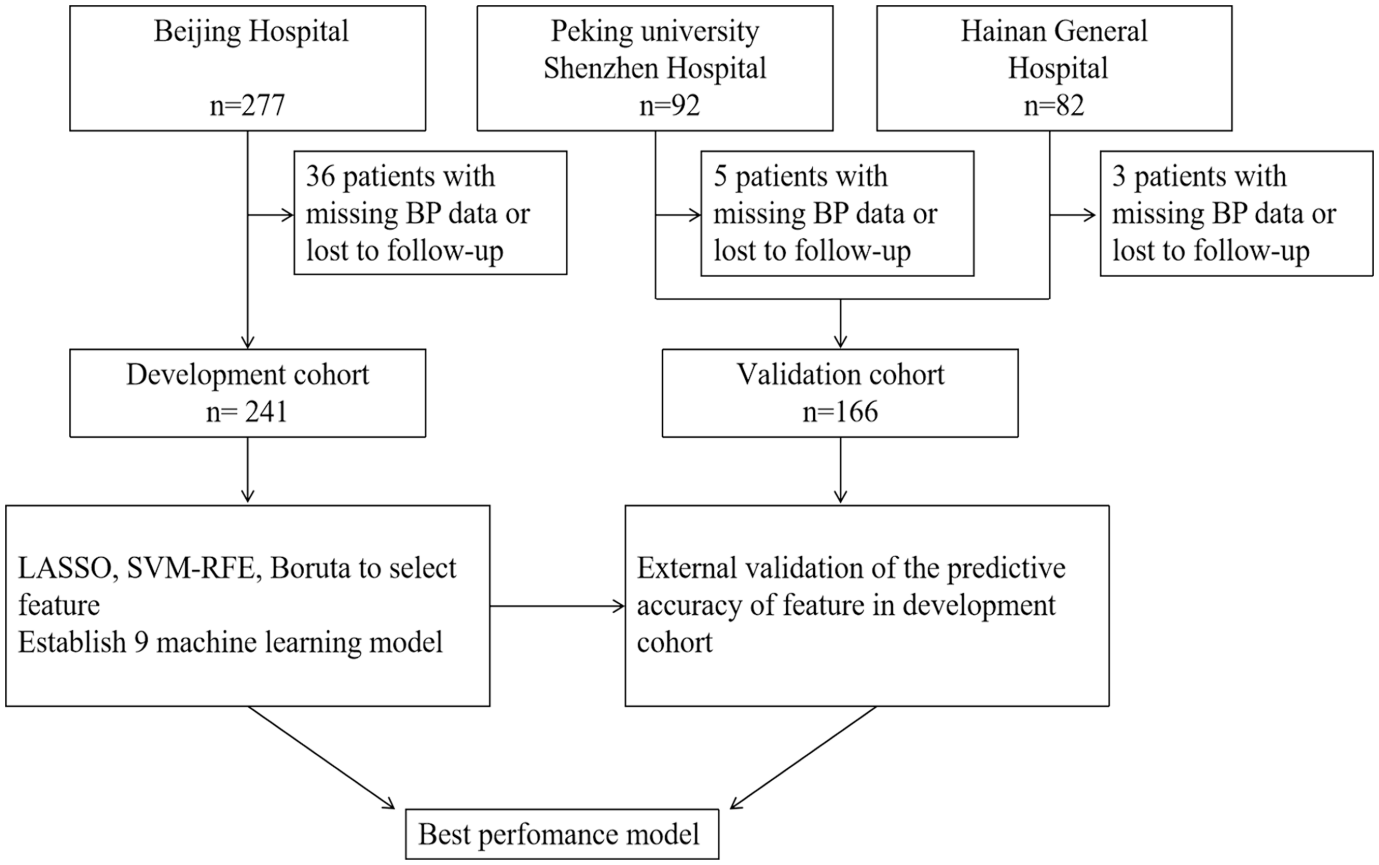
Study flow.

This retrospective, multicenter study was conducted across three hospitals in China (ChiCTR2300077202). The study flowchart was depicted in Figure 1. Patients eligible for inclusion in this study were diagnosed with AIS through either CT or MRI within 24 h of symptom onset and subsequently underwent EVT. Exclusion criteria comprised prestroke modified Rankin Scale (mRS) scores exceeding 2 and documented BP measurements exceeding more than 20% per hour within the initial 24 h. Finally, enrollment was carried out consecutively at 3 medical institutions in China: Beijing Hospital (277 cases from January 2016 to March 2023), Peking University Shenzhen Hospital (92 cases from January 2020 to March 2024), Hainan general hospital (82 cases from January 2021 to January 2023). The study was approved by Beijing Hospital Ethics Committee (2023BJYYEC-364-01).

### Data collection

A comprehensive collection of baseline clinical data was obtained from the hospital medical records system. The information collected includes basic demographic details, (age and sex), medical histories [diabetes, dyslipidemia, hypertension, atrial fibrillation (AF), coronary atherosclerotic heart disease (CHD)], and present use of antithrombotic drugs, NIHSS score at onset, occlusion site, and intravenous tissue-type plasminogen activator (IV-tPA) therapy. Recanalization status was assessed using a modified cerebral infarction thrombolysis (mTICI) grading system, where an mTICI score of 2b or 3 defined successful recanalization. The primary outcome variable that determined the patient’s 90-day functional status using the modified Rankin Scale (mRS). Good functional outcomes were defined by mRS Scores on a scale of 0 to 2, while poor outcomes were scored on a scale of 3 or higher. The mRS Scores were assessed by experienced neurologists, mainly during scheduled outpatient consultations. In cases where direct assessment was impractical, scores were obtained by telephone with the patient’s relative or caregiver.

### Blood pressure parameters

The threshold for SBP follows guidelines, the specific target is determined by the physician performing EVT. The BP monitoring was measured hourly in the ward after endovascular thrombectomy (EVT) and recorded for at least the first 24 h after surgery. BP readings were systematically recorded using Philips equipment and sphygmomanometer cuffs and transmitted to the nurse station terminal via a manual/bedside non-invasive BP monitor. BP assessments at all three participating centers used a combination of manual and automated methods to ensure comprehensive and accurate readings. As SBP was the predominant risk factor in the elderly (15–17), the study mainly focused on SBP.

To further investigate the circadian rhythm of BP, nighttime and daytime BP recordings were distinguished. Nighttime BP was recorded between 10 pm and 4 am, while daytime BP was measured between 8 am and 6 pm. These indicators provide a comprehensive understanding of the key dynamic BP fluctuations during the first 24 h after EVT.

### Model development and validation

To reduce model complexity and the risk of overfitting, as well as optimize training speed, we performed least absolute shrinkage and selection operator (LASSO), support vector machine-recursive feature elimination (SVM-RFE), and Boruta to select potential features. SVM-RFE aims to find an optimal subset of features by iteratively removing the least important features based on their weights or rankings obtained from the SVM model, ensuring a global optimal solution (18). The Boruta algorithm provides an importance score for each feature, which helps to understand the relative importance between features (19). The intersection of the selected features from these methods was utilized as the model variables. We selected 15 features, including age, sex, BMI, hypertension, CHD, diabetes, dyslipidemia, AF, stroke history, smoking history, present antithrombotic therapy, IV-tPA, NIHSS on admission, mTICI grade and occluded site. In this study, cases from Beijing hospital (BJH) cohort were utilized for model development, while the Peking University Shenzhen hospital- Hainan general hospital (PKUSZ-HN) cohorts served as an external validation set. The aim of external validation was further assessed the generalizability and predictive efficacy of the selected models. In order to evaluate the optimal predictive performance and predictive effectiveness of different SBP parameters, we constructed nine learning models, including eXtreme Gradient Boosting (XGboost), Logistic regression (LR), Decision Tree (DT), Adaptive boost (AdaBoost), GaussianNB (GNB), Gradient Boosting Decision Tree (GBDT), Multi-layer Perceptron (MLP), Support Vector Machine (SVM), and K-Nearest Neighbor Machine (KNN). In addition, we report several parameters related to model performance in this study, including area under subject operating characteristic curve (AUC), sensitivity, specificity, and accuracy. After comparing the performance of nine different ML models, the final model was determined based on the highest AUC value.

### Statistics analysis

To assess the normality of the data distribution, a Kolmogorov–Smirnov test was first conducted. Patients with missing SBP data exceeding four time points were excluded, while for other cases with missing SBP data, imputation was performed using the average of the 24-h SBP values. Descriptive statistics were used to summarize baseline characteristics and outcomes. Categorical variables were expressed in numbers and percentages, and chi-square tests were used to identify significant differences between risk factors and clinical outcomes. The normally distributed continuous variables were expressed as mean ± standard deviation (SD), and the comparison was performed using a *T*-test. All statistical analyses were performed using SPSS v25 (IBM Corporation, NY), and an α-level of 0.05 was adopted as the threshold for significance.

## Results

### Patients characteristics

A total of 451 AIS patients aged above 18 underwent EVT treatment. Forty-four patients were excluded due to missing BP data or loss to follow-up, with 36 excluded from the development group and 8 from the external cohort. Ultimately, 407 patients were included in the analysis (Figure 1), comprising 241 in the development group and 166 in the validation group. Baseline characteristics of the patients were shown in Table 1. The median age of these patients was 70 years, with 253 males (62.2%). The median baseline NIHSS score was 14 (range: 9–19), with 129 (31.7%) receiving IV-tPA treatment before EVT. One hundred ninety-six patients (81.3%) and 132 patients (79.5%) achieved successful recanalization in the two cohorts, respectively. Finally, 182 patients (44.7%) had favorable outcomes. Missing values were imputed for BP readings (*n* = 27), BMI (*n* = 43), atrial fibrillation (*n* = 1), diabetes (*n* = 1), hypertension (*n* = 1), dyslipidemia (*n* = 1), smoking history (*n* = 1), IV-tPA (*n* = 1), coronary heart disease (*n* = 1), antithrombotic therapy (*n* = 18), and stroke history (*n* = 1). The average daytime and nighttime SBP values for the population before and after imputation were both 131 mmHg (*SD*: 14.5) and 127 mmHg (*SD*: 14.5), respectively. In the development group, the postoperative daytime, nighttime, and 24-h BPs were mm 133, 129, and 131 mmHg, respectively, while in the validation group, they were 128, 125, and 127 mmHg, respectively.

**Table 1:** Baseline characteristics of patients in BJH and PKUSZ-HN cohorts.

	BJH cohort (development set)			PKUSZ-HN cohort (validation set)		
Variable	Favorable	Unfavorable	Total	Favorable	Unfavorable	Total
Sex, male (%)	77 (63.6)	69 (57.5)	146 (60.6)	40 (65.6)	67 (63.8)	107 (64.5)
Age, median (IQR)	71 (61–80)	76.5 (66–83)	73 (62–82)	65 (52–72)	67 (60.5–74.5)	66 (58–74)
BMI, mean (SD)	24.6 (4.8)	24.6 (5.6)	24.6 (5.2)	23.8 (4.5)	24.2 (4.1)	24.1 (4.2)
Hypertension (%)	82 (67.8)	101 (84.2)	183 (75.9)	42 (68.9)	70 (66.7)	112 (67.5)
Stroke (%)	35 (28.9)	44 (36.7)	79 (32.8)	16 (26.2)	18 (17.1)	34 (20.5)
Atrial fibrillation (%)	36 (29.8)	52 (43.3)	88 (36.5)	18 (29.5)	41 (39.0)	59 (35.5)
CHD (%)	26 (21.5)	53 (44.2)	79 (32.8)	9 (14.8)	22 (21.0)	31 (18.7)
Diabetes (%)	44 (36.4)	50 (41.7)	94 (39.0)	13 (21.3)	27 (25.7)	40 (24.1)
Antithrombotic drug use (%)	35 (28.9)	36 (30.0)	71 (29.5)	18 (29.5)	34 (32.4)	52 (31.3)
Anterior circulation (%)	103 (85.1)	94 (78.3)	197 (81.7)	55 (90.2)	82 (78.1)	137 (82.5)
Smoke (%)	47 (38.8)	37 (30.8)	84 (34.9)	15 (24.6)	34 (32.4)	49 (29.5)
Dyslipidemia (%)	44 (36.4)	49 (40.8)	93 (38.6)	6 (9.8)	11 (10.5)	17 (10.2)
IV-tPA (%)	25 (20.7)	38 (31.7)	63 (26.1)	31 (50.8)	35 (33.3)	66 (39.8)
NIHSS, median (IQR)	9 (6–15)	14.5 (10.5–20)	13 (8–18)	13 (9–18)	19.5 (12–27.5)	16 (11–23)
Successful recanalization (%)	107 (88.4)	89 (74.2)	196 (81.3)	59 (96.7)	73 (69.5)	132 (79.5)
BP (SD)						
Daytime SBP	131 (13.8)	134 (16.0)	133 (15.0)	124 (12.5)	130 (14.2)	128 (13.9)
Nighttime SBP	126 (14.4)	132 (16.1)	129 (15.6)	120 (10.5)	129 (13.0)	125 (12.9)
24-h SBP	129 (12.8)	133 (14.6)	131 (13.9)	123 (10.4)	130 (11.6)	127 (11.7)

BMI, body mass index; CHD, coronary atherosclerotic heart disease; SBP, systolic blood pressure.

### Feature selection

**Figure 2:**
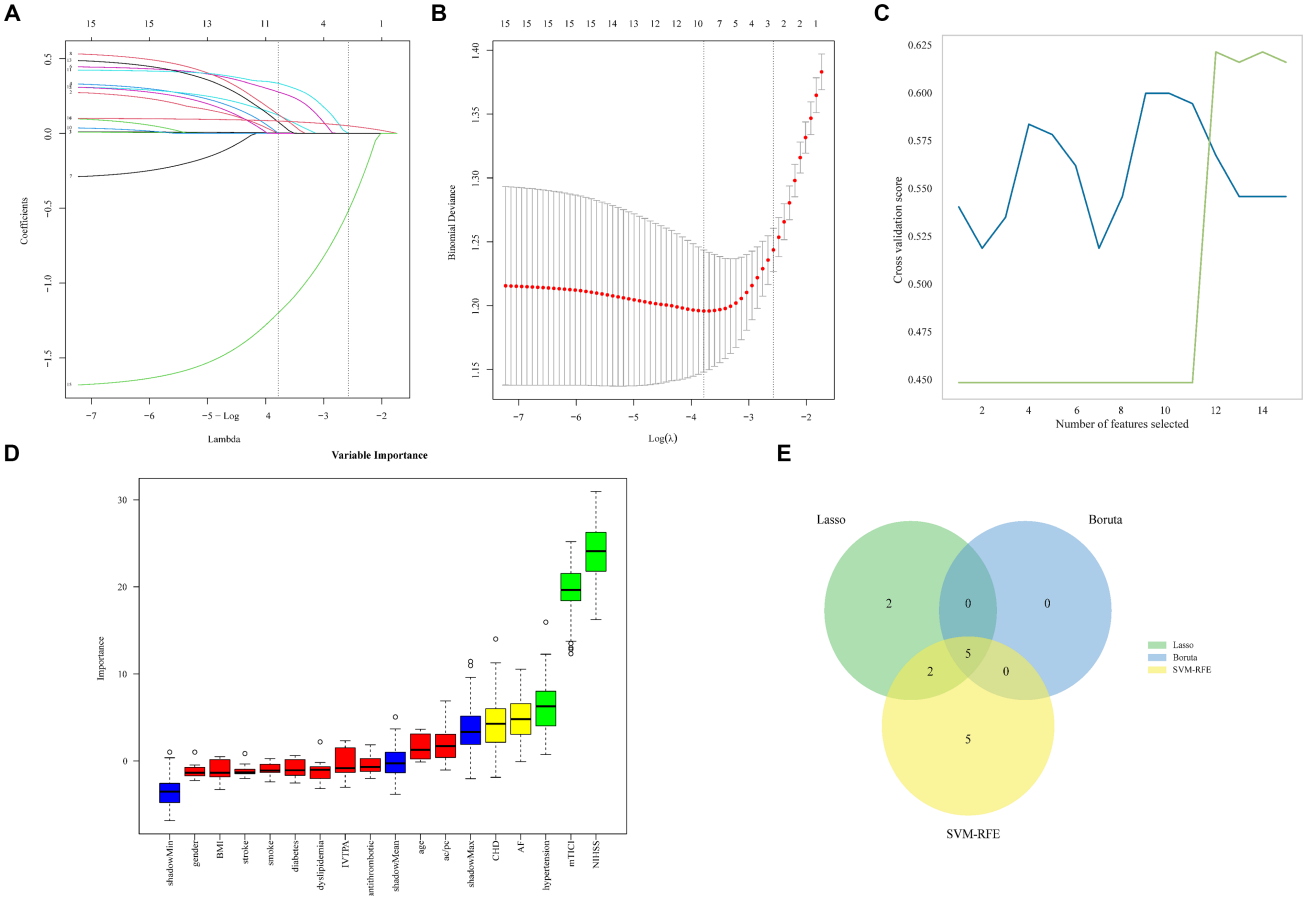
Machine learning identified crucial variable for 3-month functional outcome in AIS. Five genes that 146 were most suitable for diagnosis in the LASSO model were identified **(A,B)**. The SVM-RFE algorithm screened 12 variables **(C)**. The Boruta algorithm identified 5 variables. The green, yellow and red box plots are significant, tentative and rejected variables. The variables here include significant and tentative variables which may improve consensus identification of key variables **(D)**. Venn diagram showing the intersection of the three algorithms **(E)**.

Three machine learning algorithms, LASSO, SVM-RFE, and Boruta, were performed to identify and select potential variables associated with the 3-month functional outcome (Figure 2). The LASSO analysis identified potential prognostics factors as NIHSS, mTICI grade, CHD, diabetes, hypertension, AF, IV-tPA, age and BMI, while SVM-RFE determined gender, IV-tPA, AF, hypertension, dyslipidemia, diabetes, stroke, CHD, antithrombotic therapy, occluded site, mTICI grade, NIHSS, and Boruta established NIHSS, mTICI, CHD, AF, hypertension. By selecting the variables overlapped among the three algorithms, the final variables for modeling were determined as NIHSS, mTICI, CHD, AF, hypertension as potential factors.

### Model validation

**Figure 3:**
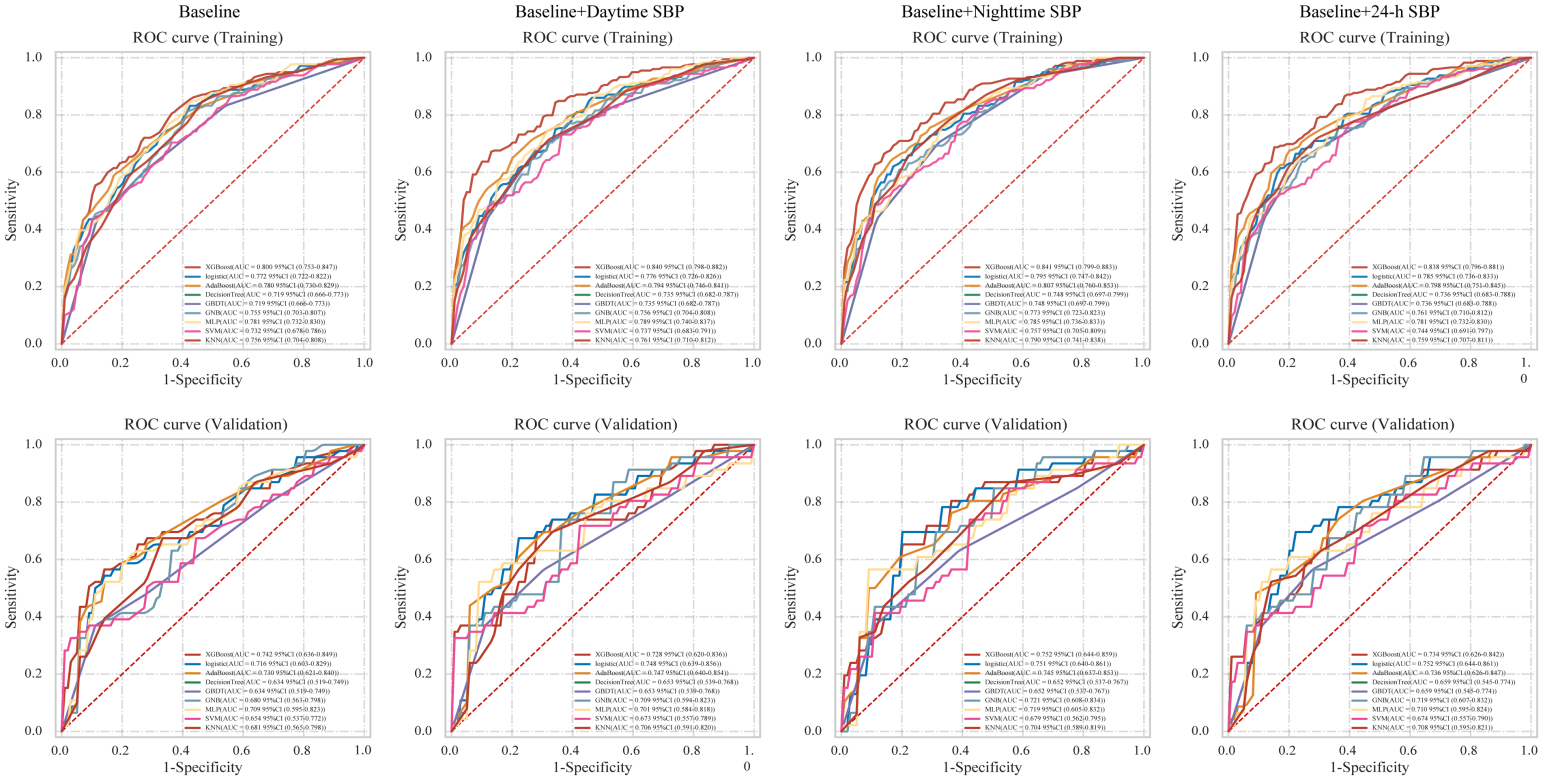
Receiver-operating characteristic curves for nine machine learning models. LR, logistics regression; XGBoost, eXtreme Gradient Boosting; Adaboost, Adaptive Boosting; DT, decision tree; GBDT, Gradient Boosting Decision Tree; GNB, Gaussian Naïve Bayes; MLP, multi-layer perceptron neural network; SVM, support vector machine; KNN, K-Nearest Neighbor Machine.

After determining the final 5 variables, we applied 9 machine learning algorithms to establish model and evaluated performance (Figure 3). The specific parameters of different machine learning methods were shown in Supplementary Table S1. The AUC of all algorithms increased to varying degrees after the addition of SBP-related parameters. The AUC of LR algorithm in the baseline model was 0.772, and after adding daytime BP, nighttime BP, and 24-h BP, the AUCs were 0.776, 0.795, and 0.785, respectively. The AUC of XGboost algorithm in the baseline model was 0.800, and AUC were 0.841, 0.840 and 0.838 after adding daytime BP, night BP and 24-h BP, respectively. The evaluation indicators of models were shown in Table 2. Among all algorithms, XGBoost with baseline plus nighttime SBP demonstrated the best performance in terms of AUC, with accuracy of 0.751, specificity of 0.863, and sensitivity of 0.751. Other evaluation metrics for the models are presented in Table 2.

**Table 2:** Model performance in development cohort.

Algorithm	Model	AUC	Cutoff	Accuracy	Sensitivity	Specificity	PPV	NPV	F1-Score
LR	Baseline	0.772 (0.722–0.822)	0.439	0.714	0.827	0.582	0.707	0.726	0.762
	Baseline + Nighttime SBP	0.795 (0.747–0.842)	0.561	0.720	0.698	0.753	0.775	0.667	0.735
	Baseline + Daytime SBP	0.776 (0.726–0.826)	0.499	0.705	0.743	0.664	0.729	0.674	0.736
	Baseline + 24 h SBP	0.785 (0.736–0.833)	0.620	0.711	0.615	0.836	0.820	0.635	0.702
XGBoost	Baseline	0.800 (0.753–0.847)	0.530	0.717	0.721	0.733	0.767	0.667	0.743
	Baseline + Nighttime SBP	0.841 (0.799–0.883)	0.657	0.751	0.665	0.863	0.855	0.674	0.748
	Baseline + Daytime SBP	0.840 (0.798–0.882)	0.657	0.754	0.637	0.904	0.890	0.667	0.742
	Baseline + 24 h SBP	0.838 (0.796–0.881)	0.610	0.757	0.687	0.849	0.847	0.685	0.759
AdaBoost	Baseline	0.780 (0.730–0.829)	0.522	0.695	0.598	0.822	0.808	0.621	0.687
	Baseline + Nighttime SBP	0.807 (0.760–0.853)	0.525	0.717	0.642	0.836	0.843	0.636	0.729
	Baseline + Daytime SBP	0.794 (0.746–0.841)	0.508	0.711	0.648	0.801	0.797	0.643	0.715
	Baseline + 24 h SBP	0.798 (0.751–0.845)	0.513	0.726	0.676	0.801	0.808	0.659	0.736
DT	Baseline	0.719 (0.666–0.773)	0.566	0.637	0.603	0.726	0.821	0.561	0.696
	Baseline + Nighttime SBP	0.748 (0.697–0.799)	0.615	0.637	0.704	0.678	0.821	0.561	0.758
	Baseline + Daytime SBP	0.735 (0.682–0.787)	0.647	0.637	0.682	0.719	0.821	0.561	0.745
	Baseline + 24 h SBP	0.736 (0.683–0.788)	0.685	0.637	0.642	0.767	0.821	0.561	0.721
GBDT	Baseline	0.719 (0.666–0.773)	0.581	0.637	0.603	0.726	0.821	0.561	0.696
	Baseline + Nighttime SBP	0.748 (0.697–0.799)	0.674	0.637	0.704	0.678	0.821	0.561	0.758
	Baseline + Daytime SBP	0.735 (0.682–0.787)	0.728	0.637	0.682	0.719	0.821	0.561	0.745
	Baseline + 24 h SBP	0.736 (0.683–0.788)	0.784	0.637	0.642	0.767	0.821	0.561	0.721
GNB	Baseline	0.755 (0.703–0.807)	0.380	0.708	0.804	0.596	0.708	0.707	0.753
	Baseline + Nighttime SBP	0.773 (0.723–0.823)	0.529	0.695	0.603	0.815	0.799	0.623	0.687
	Baseline + Daytime SBP	0.756 (0.704–0.808)	0.384	0.702	0.771	0.623	0.714	0.684	0.741
	Baseline + 24 h SBP	0.761 (0.710–0.812)	0.474	0.692	0.637	0.767	0.769	0.629	0.697
MLP	Baseline	0.780 (0.730–0.829)	0.451	0.723	0.844	0.582	0.711	0.746	0.772
	Baseline + Nighttime SBP	0.784 (0.735–0.833)	0.523	0.717	0.726	0.712	0.754	0.675	0.740
	Baseline + Daytime SBP	0.777 (0.727–0.827)	0.481	0.714	0.760	0.664	0.734	0.688	0.747
	Baseline + 24 h SBP	0.768 (0.717–0.819)	0.523	0.723	0.760	0.685	0.746	0.694	0.753
SVM	Baseline	0.732 (0.678–0.786)	0.546	0.665	0.704	0.637	0.701	0.623	0.703
	Baseline + Nighttime SBP	0.757 (0.705–0.809)	0.500	0.695	0.777	0.603	0.706	0.679	0.740
	Baseline + Daytime SBP	0.737 (0.683–0.791)	0.517	0.689	0.732	0.644	0.714	0.657	0.723
	Baseline + 24 h SBP	0.744 (0.691–0.797)	0.488	0.692	0.771	0.603	0.703	0.677	0.735
KNN	Baseline	0.756 (0.704–0.808)	0.444	0.689	0.849	0.534	0.695	0.680	0.764
	Baseline + Nighttime SBP	0.790 (0.741–0.838)	0.556	0.677	0.654	0.781	0.798	0.602	0.719
	Baseline + Daytime SBP	0.761 (0.710–0.812)	0.500	0.680	0.715	0.678	0.770	0.613	0.741
	Baseline + 24 h SBP	0.759 (0.707–0.811)	0.500	0.702	0.721	0.705	0.793	0.632	0.755

LR, logistics regression; XGBoost, eXtreme Gradient Boosting; Adaboost, Adaptive Boosting; DT, decision tree; GBDT, Gradient Boosting Decision Tree; GNB, Gaussian Naïve Bayes; MLP, multi-layer perceptron neural network; SVM, support vector machine; KNN, K-Nearest Neighbor Machine.

To provide a better understanding of the effects of different models, further validation was conducted in the external validation set (Table 3). Similarly, after adding different BP parameters, the performance of all models improved in the validation set. The AUC of XGboost algorithm in baseline model is 0.742, 0.728, 0.752, 0.734, respectively, after increasing daytime BP, night BP and 24-h BP, respectively. For Adaboost and KNN algorithms in the baseline plus nighttime SBP model, the AUCs were 0.745 and 0.704, with accuracy, sensitivity, and specificity of 0.695, 0.500, 0.917, and 0.634, 0.870, 0.444, respectively. In summary, adding SBP levels in the predictive model established by 9 algorithms can improve AUC, with nighttime SBP showing the most significant improvement. XGBoost emerged as the best-performing algorithm.

**Table 3:** Model performance in validation cohort.

Algorithm	Model	AUC	Cutoff	Accuracy	Sensitivity	Specificity	PPV	NPV	F1-Score
LR	Baseline	0.716 (0.603–0.829)	0.439	0.610	0.543	0.861	0.635	0.567	0.586
	Baseline + Nighttime SBP	0.751 (0.640–0.861)	0.561	0.744	0.696	0.806	0.821	0.674	0.753
	Baseline + Daytime SBP	0.748 (0.639–0.856)	0.499	0.695	0.674	0.778	0.744	0.641	0.707
	Baseline + 24 h SBP	0.752 (0.644–0.861)	0.620	0.671	0.696	0.778	0.806	0.588	0.747
XGBoost	Baseline	0.742 (0.636–0.849)	0.530	0.683	0.565	0.861	0.750	0.619	0.645
	Baseline + Nighttime SBP	0.752 (0.644–0.859)	0.657	0.72	0.652	0.806	0.811	0.644	0.723
	Baseline + Daytime SBP	0.728 (0.620–0.836)	0.657	0.659	0.696	0.722	0.750	0.587	0.722
	Baseline + 24 h SBP	0.734 (0.626–0.842)	0.610	0.671	0.783	0.639	0.744	0.605	0.763
AdaBoost	Baseline	0.730 (0.621–0.840)	0.522	0.659	0.587	0.806	0.781	0.580	0.670
	Baseline + Nighttime SBP	0.745 (0.637–0.853)	0.525	0.695	0.500	0.917	0.800	0.617	0.615
	Baseline + Daytime SBP	0.747 (0.640–0.854)	0.508	0.683	0.587	0.806	0.778	0.609	0.669
	Baseline + 24 h SBP	0.736 (0.626–0.847)	0.513	0.659	0.478	0.917	0.725	0.595	0.576
DT	Baseline	0.634 (0.519–0.749)	0.566	0.598	0.370	0.889	0.810	0.525	0.507
	Baseline + Nighttime SBP	0.652 (0.537–0.767)	0.615	0.598	0.370	0.889	0.810	0.525	0.507
	Baseline + Daytime SBP	0.653 (0.539–0.768)	0.647	0.598	0.565	0.694	0.810	0.525	0.666
	Baseline + 24 h SBP	0.659 (0.545–0.774)	0.685	0.598	0.565	0.722	0.810	0.525	0.666
GBDT	Baseline	0.634 (0.519–0.749)	0.581	0.598	0.370	0.889	0.810	0.525	0.507
	Baseline + Nighttime SBP	0.652 (0.537–0.767)	0.674	0.598	0.370	0.889	0.810	0.525	0.507
	Baseline + Daytime SBP	0.653 (0.539–0.768)	0.728	0.598	0.565	0.694	0.810	0.525	0.666
	Baseline + 24 h SBP	0.659 (0.545–0.774)	0.784	0.598	0.565	0.722	0.810	0.525	0.666
GNB	Baseline	0.680 (0.563–0.798)	0.380	0.646	0.370	0.917	0.667	0.613	0.476
	Baseline + Nighttime SBP	0.721 (0.608–0.834)	0.529	0.598	0.696	0.694	0.741	0.527	0.717
	Baseline + Daytime SBP	0.709 (0.594–0.823)	0.384	0.683	0.717	0.639	0.708	0.647	0.713
	Baseline + 24 h SBP	0.719 (0.607–0.832)	0.474	0.610	0.761	0.583	0.706	0.542	0.732
MLP	Baseline	0.707 (0.592–0.821)	0.451	0.646	0.543	0.889	0.667	0.613	0.599
	Baseline + Nighttime SBP	0.724 (0.612–0.836)	0.523	0.646	0.587	0.861	0.707	0.585	0.642
	Baseline + Daytime SBP	0.706 (0.591–0.821)	0.481	0.634	0.609	0.806	0.674	0.583	0.640
	Baseline + 24 h SBP	0.681 (0.564–0.798)	0.523	0.610	0.500	0.889	0.659	0.553	0.569
SVM	Baseline	0.654 (0.537–0.772)	0.546	0.585	0.326	0.972	0.636	0.526	0.431
	Baseline + Nighttime SBP	0.679 (0.562–0.795)	0.500	0.659	0.739	0.583	0.673	0.633	0.705
	Baseline + Daytime SBP	0.673 (0.557–0.789)	0.517	0.634	0.326	1.000	0.660	0.594	0.437
	Baseline + 24 h SBP	0.674 (0.557–0.790)	0.488	0.622	0.348	0.944	0.642	0.586	0.451
KNN	Baseline	0.681 (0.565–0.798)	0.444	0.634	0.674	0.667	0.674	0.583	0.674
	Baseline + Nighttime SBP	0.704 (0.589–0.819)	0.556	0.634	0.870	0.444	0.750	0.560	0.805
	Baseline + Daytime SBP	0.706 (0.591–0.820)	0.500	0.659	0.696	0.667	0.765	0.583	0.729
	Baseline + 24 h SBP	0.708 (0.595–0.821)	0.500	0.646	0.522	0.861	0.758	0.571	0.618

LR, logistics regression; XGBoost, eXtreme Gradient Boosting; Adaboost, Adaptive Boosting; DT, decision tree; GBDT, Gradient Boosting Decision Tree; GNB, Gaussian Naïve Bayes; MLP, multi-layer perceptron neural network; SVM, support vector machine; KNN, K-Nearest Neighbor Machine.

### Model construction and performance

**Figure 4:**
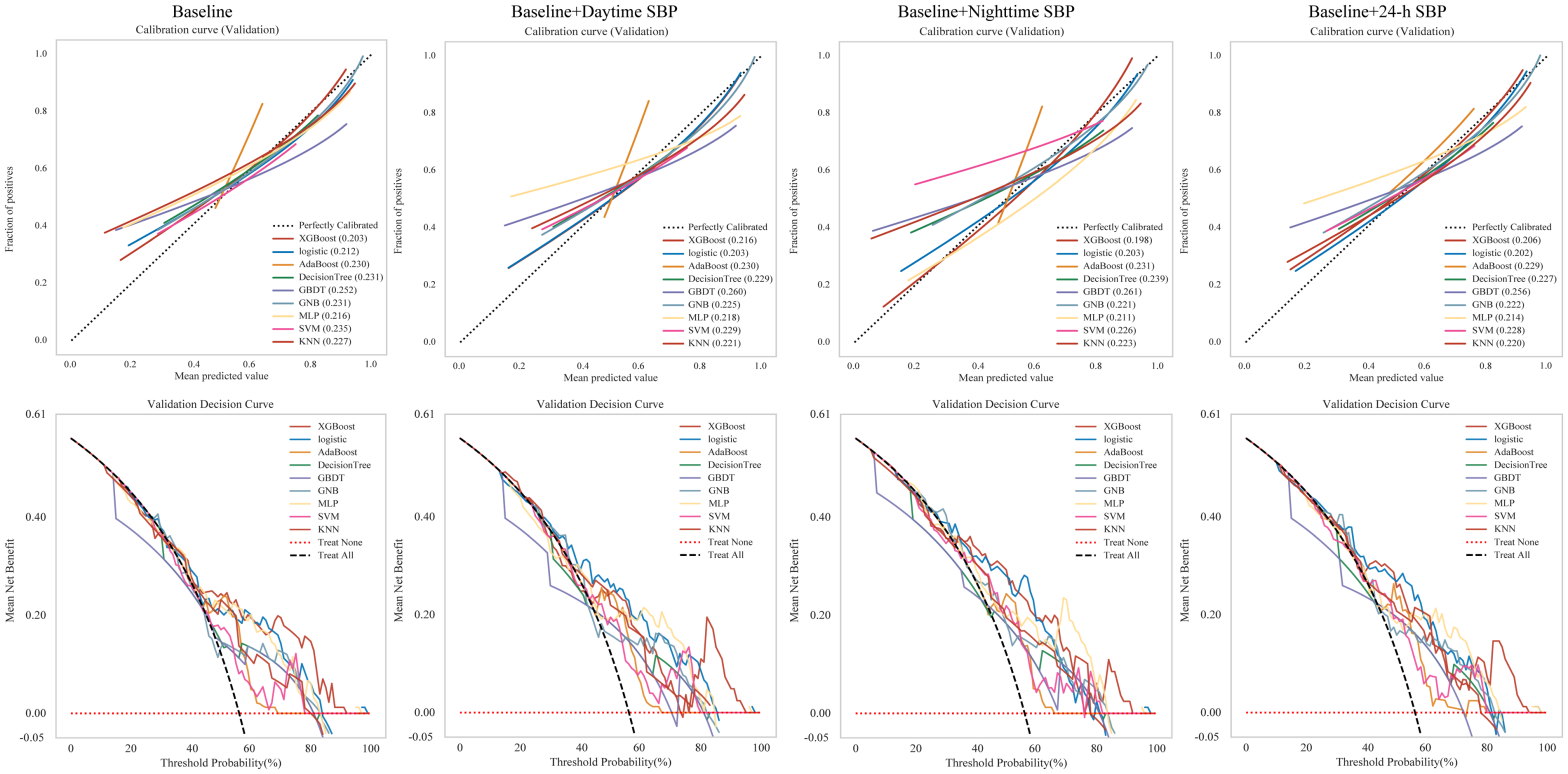
Calibration curves and Validation decision curves for nine machine learning models. LR, logistics regression; XGBoost, eXtreme Gradient Boosting; Adaboost, Adaptive Boosting; DT, decision tree; GBDT, Gradient Boosting Decision Tree; GNB, Gaussian Naïve Bayes; MLP, multi-layer perceptron neural network; SVM, support vector machine; KNN, K-Nearest Neighbor Machine.

To assessed the bias and clinical benefit of the different models, we applied the DCA curve and calibration curve to evaluate the models generated by nine algorithms (Figure 4). In the models of baseline and baseline + nighttime SBP, XGboost showed the lowest brier score of 0.203 and 0.198, respectively, demonstrating the robustness compared to the other algorithmic models. The logistic algorithm exhibited the highest robustness in the baseline + daytime SBP and baseline +24-h SBP models, with scores of 0.203 and 0.202, respectively, followed by the XGboost algorithm, with scores of 0.216 and 0.206, respectively.

## Discussion

The blood pressure levels post-AIS are strongly correlated with functional outcome, however there were no studies on the association between circadian BP patterns and clinical outcome after EVT. This study firstly investigated the association between the circadian BP patterns in the first consecutive 24 h post-EVT and functional outcomes, and to evaluate risk prognosis using different BP parameters. Compared with daytime SBP and 24-h SBP, nighttime SBP provided more predictive information regarding functional outcome in different machine learning algorithms. The XGBoost model outperformed all other ML methods in discrimination and accuracy, with AUC values of 0.841 in the development set and 0.752 in the validation set. Therefore, nighttime SBP may offer a better approach to utilizing BP assessment for predicting AIS outcomes.

Previous population and clinical studies have demonstrated a correlation between BP levels and the prognosis of ischemic stroke (20, 21). Several observational studies have elucidated the association of higher BP with poorer clinical outcomes (22). For instance, Matusevicius et al. (23) reported a correlation between BP and the incidence of symptomatic intracerebral hemorrhage. Recent randomized controlled trials (RCTs) have suggested adverse effects of intensive blood pressure lowering (24, 25). One possible explanation for this was that blood pressure was considered a comprehensive reflection of stroke rather than a direct cause of prognosis. Post-EVT BP control after AIS needs to be tailored according to individual patient conditions, although there is currently no consensus on the optimal goals of post-stroke blood pressure. In our study, the postoperative BP of patients was not strictly uniformly after across different centers, but all with successful recanalization maintained BP levels below <140 mmHg. The BP levels in both cohorts showed a strong correlation with functional prognosis.

Recent studies suggest that nighttime BP may be a more relevant predictor of all-cause mortality and composite cardiovascular outcome (10, 11), and associated with a higher incidence of stroke, but the relationship between nighttime BP and AIS prognosis is currently unknown. Normally, BP fluctuates throughout the day with a circadian rhythm, typically peaking in the morning and afternoon. Therefore, the relationship between circadian BP patterns and prognosis may be a question worth exploring. Previous studies identified SBP before reperfusion therapy as a key prognostic factor in AIS patients undergoing intravenous thrombolysis or EVT treatment. They utilized logistic regression algorithm to construct a predictive model for 3-month functional outcomes, achieving an AUC of 0.865 in development cohort and an AUC of 0.779 in external cohort (26). In this study, predictive models of functional outcome were constructed based on BP rhythm during different time periods, primarily comparing the relationships between daytime BP, nighttime BP, and 24-h average BP and prognosis. We performed nine machine learning models to ensure coverage of various modeling principles and strategies in machine learning, including decision trees, ensemble learning, probabilistic models, neural networks, and distance-based methods. By comparing the performance of these methods, we can objectively evaluate the performance of various algorithms, avoiding biases associated with a single method and providing a more robust basis for selection. Our findings indicated that among all models constructed, including LR and other machine learning algorithms, nighttime BP demonstrates the highest predictive power compared to daytime BP and 24-h average BP. This suggests that nighttime blood pressure levels more accurately reflect the potential functional prognosis of patients after EVT in AIS. Although increasing evidence suggests that blood pressure variability (BPV) is a more meaningful indicator than absolute blood pressure values (27–29), unfortunately, most BP variability parameters are not available in a timely manner, thereby affecting their practical applicability. Therefore, nighttime SBP may provide more clinical information for the prognosis of AIS patients after EVT. Possible explanation of superiority of the nighttime SBP compared to other SBP parameters may be attribute to more standardized measurement methods and increased activity during daytime. Nighttime BP is a more standardized measurement, Clinical practice should focus on nighttime SBP readings in AIS patients after EVT with its readings reflecting the true basal blood pressure levels of patients.

There were some limitations in this study. Firstly, it was a retrospective non-randomized observational cohort study, and potential biases may exist across different centers in patient selection, clinical practices, and procedural techniques. Secondly, some key variable in this study, such as baseline Aspects score, onset time, and collateral status, were not included. Thirdly, information regarding antihypertensive medications was not documented.

## Conclusion

In conclusion, compared to daytime SBP and 24-h SBP, nighttime SBP provided more prognostic information following AIS EVT treatment. Therefore, nighttime SBP should be considered as the optimal measurement for assessing the prognosis of AIS patients.

## Data availability statement

The original contributions presented in the study are included in the article/Supplementary material, further inquiries can be directed to the corresponding authors.

## Ethics statement

The studies involving humans were approved by the Beijing Hospital Ethics Committee. The studies were conducted in accordance with the local legislation and institutional requirements. The ethics committee/institutional review board waived the requirement of written informed consent for participation from the participants or the participants' legal guardians/next of kin due to the retrospective nature of the study.

## Author contributions

DX: Conceptualization, Formal analysis, Investigation, Methodology, Validation, Writing – original draft, Writing – review & editing. PQ: Data curation, Supervision, Validation, Writing – review & editing. PL: Validation, Writing – review & editing. HY: Resources, Validation, Writing – review & editing. GFY: Resources, Validation, Writing – review & editing. DS: Data curation, Writing – review & editing. SL: Data curation, Writing – review & editing. GZY: Data curation, Writing – review & editing. JD: Data curation, Writing – review & editing. HL: Data curation, Funding acquisition, Resources, Validation, Writing – review & editing. HQ: Data curation, Resources, Validation, Writing – review & editing. DW: Funding acquisition, Project administration, Resources, Supervision, Writing – review & editing. JL: Funding acquisition, Resources, Supervision, Writing – review & editing.
