# Supplementary material for: Machine learning models reveal the critical role of nighttime systolic blood pressure in predicting functional outcome for acute ischemic stroke after endovascular thrombectomy
Source: Front Neurol. 2024 May 9;15:1405668. doi: 10.3389/fneur.2024.1405668 (PMC11112097; doi:10.3389/fneur.2024.1405668)
Supplement: Supplementary file 1 [file Table_1.DOCX]

**Supplemental Table 1. Specific parameters of different machine learning methods**

| **Xgboost model parameters**  **Objective:** binary:logistic  **learning_rate:** 0.1  **max_depth:** 4  **min_child_weight:** 10  **reg_lambda:** 1 | **LogisticRegression**  **C:** 1  **max_iter**: 100  **penalty**: l2  **tol**: 0.0001 |
| --- | --- |
| **DecisionTreeClassifier**  **criterion:** gini  **max_depth:** 20  **min_samples_leaf:** 50  **min_samples_split:** 50 | **AdaBoostClassifier**  **learning_rate**: 0.1  **n_estimators**: 40 |
| **GradientBoostingClassifie**  **learning_rate**: 2  **loss**: log_loss  **max_depth**: 20  **min_samples_leaf**: 50  **min_samples_split**: 2  **n_estimators**: 1 | **GaussianNB:**  **priors:** None  **var_smoothing:** 0.01 |
| **SVC**  **C**: 1  **kernel**: rbf  **tol**: 0.001 | **MLPClassifier**  **activation:** relu  **hidden_layer_sizes:** (60, 10)  **max_iter:** 200 |
| **KNeighborsClassifier**  **n_neighbors**: 18  **weights**: uniform |  |
